# Performance Analysis on the Blast Resistance of Hybrid-Reinforced Concrete Walls

**DOI:** 10.1021/acsomega.4c10604

**Published:** 2025-03-26

**Authors:** Casim Yazici, Fatih Mehmet Özkal

**Affiliations:** † Department of Construction, Aǧrı İbrahim Çeçen University, Aǧrı 04400, Türkiye; ‡ Department of Civil Engineering, Atatürk University, Erzurum 25240, Türkiye

## Abstract

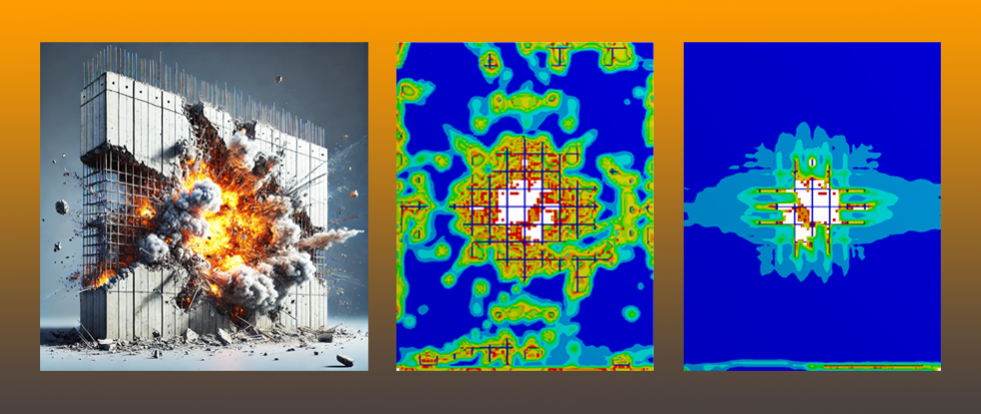

This study investigates the blast resistance of reinforced concrete (RC) shear walls reinforced with hybrid configurations of traditional steel rebars and advanced fiber-reinforced polymers (FRPs), including carbon fiber reinforced polymer (CFRP), glass fiber reinforced polymer (GFRP), and basalt fiber reinforced polymer (BFRP). A validated finite element analysis (FEA) model was used to evaluate ten reinforcement configurations subjected to central contact explosive loads. RC shear walls measuring 2100 mm × 2800 mm × 300 mm were analyzed based on displacement, surface damage, and energy absorption. Configurations fully reinforced with CFRP demonstrated the highest blast resistance, effectively minimizing displacement and surface damage due to CFRP’s superior stiffness and tensile strength. BFRP configurations exhibited higher energy dissipation but resulted in increased deformation and damage, while GFRP configurations provided intermediate performance, balancing stiffness and flexibility. Hybrid configurations combining steel and FRPs offered an effective compromise by optimizing energy absorption, structural integrity, and cost-effectiveness. These findings highlight CFRP as the optimal material for high-impact applications, while hybrid and material-specific reinforcements provide adapted solutions for moderate resistance requirements or environmental constraints. This study emphasizes the importance of material selection and reinforcement strategies in the design of durable, blast-resistant RC structures.

## Introduction

Recent advancements in the design criteria for reinforced concrete (RC) in blast-hardened structures emphasize an improved response to explosive loads, leading to safer and more cost-effective protective shelters and barriers. These updates primarily involve increases in allowable design stresses and ultimate flexural deflections under shock loads, significantly enhancing the energy absorption capacity of conventional reinforced concrete. With these revisions, conventionally reinforced concrete can now be designed to accommodate deflections up to four times greater than previously allowed, reducing the dependency on more expensive reinforcement solutions, such as laced reinforced concrete. These improvements represent a significant step forward in optimizing the performance and cost-effectiveness of protective structures under extreme loading conditions.^1^

Reinforced concrete structures face significant challenges at elevated temperatures, which might be induced by explosive loads. A significant threat to all structural members is possible, leading to the loss of life and property. While steel-reinforced structures exhibit excellent performance at room temperature, their metallurgical composition causes a substantial reduction in strength when exposed to high temperatures.^2,3^ A recent study explored the structural behavior of reinforced concrete beams exposed to temperatures up to 800 °C, providing insights into their postexposure performance. The results demonstrated significant reductions in concrete compressive strength and steel yield strength after 600 °C, alongside obvious degradation in load-bearing capacity and bond performance between concrete and steel. Structural deterioration significantly impacts the safety and performance of reinforced concrete members.^4^ Previous studies highlight the importance of accurate modeling to assess fire effects, focusing on thermal and structural interactions including the bond behavior between concrete and rebar. These findings emphasize the critical importance of incorporating high-temperature resistance considerations into the design and reinforcement strategies of concrete structures to ensure safety and durability under extreme conditions.^5^

Reinforced concrete structures have long been a cornerstone of the construction industry due to their robustness, adaptability, and cost-effectiveness. However, as the demand for more durable and sustainable infrastructure grows, the limitations of traditional steel reinforcement in concrete structures are becoming increasingly evident. Durability can be assessed through various parameters, including permeability, porosity, alkali-silica reaction mitigation, resistance to freeze–thaw cycles, resistance to wet–dry cycles, elevated-temperature performance, resistance to chemical attacks, corrosion resistance, abrasion resistance, and impact resistance among others.^6,7^ Over time, miscellaneous effects can severely compromise the structural integrity of concrete, leading to frequent maintenance, repairs, or even replacement, which increases the overall cost and environmental impact of construction.^8^ In response to these challenges, fiber-reinforced polymer (FRP) reinforcements have emerged as a promising alternative, offering high corrosion resistance, exceptional tensile strength, and lightweight properties. These advantages not only improve the longevity of concrete structures but also reduce the need for costly maintenance and repairs, especially in challenging environmental conditions.^9^

Among FRP types, glass fiber-reinforced polymer (GFRP), carbon fiber-reinforced polymer (CFRP), and basalt fiber-reinforced polymer (BFRP) have gained particular attention in structural engineering due to their unique mechanical and physical properties.^10^ GFRP is valued for its cost-effectiveness and corrosion resistance, making it particularly suitable for structures exposed to seawater and chemically aggressive environments. However, GFRP has a lower elastic modulus than steel, which can lead to increased deformation and larger crack widths under heavy loads, necessitating specific design modifications to mitigate these effects and ensure adequate structural performance.^11^ Despite these limitations, GFRP has demonstrated substantial benefits in terms of durability and cost savings, especially in marine and coastal environments where corrosion resistance is essential.^12^

Carbon fiber reinforced polymer (CFRP) composites are increasingly replacing metals in the marine, automotive, aerospace, and construction industries due to their exceptional mechanical strength. However, their brittleness and low toughness limit their application in structural applications. Recent research highlights the integration of super elastomeric polyurea as a matrix with carbon fibers using vacuum-assisted resin transfer molding, which significantly enhances flexural load, stiffness, and resistance to mechanical indentation. Polyurea matrix also has remarkable stress absorption and redistribution capabilities, preventing catastrophic failure even under significant deformation. While degradation analysis shows initial strength improvement due to plasticizing effects in saline environments, prolonged exposure leads to matrix-fiber debonding. These findings position viscoelastic CFRP-polyurea composites as tough, high-performance materials suitable for harsh marine environments.^13^

CFRP, in contrast, offers a higher elastic modulus and tensile strength than GFRP, making it ideal for projects requiring robust structural reinforcement, such as high-rise buildings, bridges, and other critical infrastructure. The high strength-to-weight ratio of CFRP has proven particularly advantageous in applications demanding blast and impact resistance, such as military and industrial structures.^14^ Studies have shown that CFRP reinforcement enhances the load-bearing capacity, improves durability, and reduces crack propagation, thereby extending the service life of concrete structures. This makes CFRP a preferred choice in applications where high tensile loads or severe environmental stressors are expected.^15^ Furthermore, CFRP improves the seismic performance of reinforced concrete structures, adding value in earthquake-prone regions where both flexibility and strength are critical.^16^

BFRP is a more recent addition to the FRP family, derived from natural basalt rock. Its environmental sustainability, combined with high tensile strength and corrosion resistance, has led to its increased application in structural engineering. BFRP is particularly suited for structures subjected to high axial and lateral loads such as shear walls, where both strength and deformation control are essential. Its environmentally friendly production process, which involves melting basalt rock without chemical additives, makes BFRP a more sustainable choice for the construction industry.^12^ Additionally, BFRP’s resistance to environmental degradation and its lightweight nature make it ideal for marine applications and for use in structures where reducing self-weight is beneficial. Research has shown that BFRP-reinforced structures exhibit enhanced load-bearing capacity, improved durability, and reduced susceptibility to long-term environmental damage.^17^

Despite the advantages of FRP reinforcements, a primary challenge remains: their generally lower modulus of elasticity compared to that of steel, particularly for GFRP and BFRP. This lower modulus can lead to greater deformations and larger crack widths, potentially affecting the structural performance of FRP-reinforced concrete. To address this, researchers have explored various hybrid reinforcement systems, combining FRP with traditional steel to leverage the strengths of both materials.^7^ The integration of ultrahigh-performance concrete (UHPC) with FRP reinforcements has also shown promising results, enhancing overall strength, stiffness, and durability. UHPC’s superior compressive strength and ductility complement the tensile properties of FRP, making it particularly valuable in shear wall applications and other load-bearing structures.^18^

Recent studies emphasize that hybrid FRP systems can significantly enhance the blast resistance of reinforced concrete structures. For instance, a numerical study demonstrated that hybrid FRP reinforcements, particularly those combining CFRP and GFRP, effectively improve the energy absorption and structural response of RC slabs under explosive forces.^19^ This improvement is attributed to the superior tensile strength and lightweight properties of FRP materials, which contribute to better load distribution and reduced structural damage during blast events.^20^ Moreover, hybrid systems mitigate the limitations associated with single-type FRP reinforcements, such as CFRP’s brittleness and GFRP’s lower tensile strength.^21^

The mechanical properties of BFRP have attracted particular attention due to its cost-effectiveness and excellent performance in corrosive environments, which makes it suitable for hybrid reinforcement systems.^22^ Studies indicate that BFRP offers comparable or superior performance to GFRP and CFRP in tensile strength and durability, particularly in blast-resistant applications.^23^ Integrating BFRP into hybrid systems not only boosts structural capacity but also enhances ductility and energy dissipation, which are critical for withstanding dynamic loads.^24^

Additionally, the bond behavior between FRP bars and concrete is a significant factor influencing hybrid reinforced concrete structures’ performance. Enhanced bonding techniques, such as surface treatments for GFRP and BFRP, improve interfacial shear strength, thus increasing reinforcement effectiveness.^25^ This bond performance is essential to ensure that hybrid systems effectively transfer loads and resist forces generated during blast events.^17^

In conclusion, the integration of GFRP, CFRP, and BFRP in hybrid reinforcement systems presents a viable approach to enhancing the blast resistance of concrete walls. The combined effects of these materials improve the structural performance under extreme loading conditions while addressing the limitations of individual FRP types. Future research should focus on optimizing the design and application of these hybrid systems to maximize their effectiveness in real-world structural applications, contributing to the development of a sustainable, high-performance infrastructure that can endure a wide range of environmental and structural challenges.

## Research Significance

Reinforced concrete shear walls are essential structural components designed to withstand dynamic loads, such as explosions and seismic events. These walls provide critical resistance against lateral forces, ensuring the stability and integrity of buildings and infrastructure. However, conventional steel-reinforced concrete suffers from inherent limitations, including corrosion susceptibility, brittle failure under extreme loading conditions, and limited tensile capacity, which compromise its long-term performance.^26^ In environments exposed to aggressive weather conditions or chemical exposure, steel reinforcement may deteriorate over time, necessitating costly maintenance and repairs.

The structural performance of RC shear walls plays a pivotal role in resisting lateral forces caused by seismic and blast loads. Current advancements in construction materials have led to the increased adoption of fiber-reinforced polymer (FRP) bars, either independently or in combination with conventional steel reinforcements, to improve the mechanical performance of shear walls. FRP matrices are composite materials strengthened with various types of fibers. Common types of FRPs include carbon (CFRP), glass (GFRP), and basalt (BFRP). These composites are applied to reinforced concrete beams in forms such as laminates, rods, or fibers, using bonding techniques or mechanical anchoring.^27^ However, limited research exists on hybrid configurations that combine FRP and steel bars, particularly in squat walls, which are characterized by low aspect ratios and are often subjected to high shear forces.

Shabana et al.^28^ conducted an extensive experimental study on the seismic performance of squat RC walls reinforced with GFRP bars, focusing on walls with different aspect ratios. Their findings demonstrated that GFRP-reinforced squat walls exhibit significant improvements in the lateral load capacity, energy dissipation, and reduced shear deformations. The inclusion of web reinforcement in GFRP-reinforced walls was found to enhance the lateral stability by reducing pinching effects and improving hysteretic behavior under cyclic loads. This suggests that GFRP can be a viable alternative to steel in seismic applications, particularly when corrosion resistance is a critical design consideration.

Complementing this study, Chetchotisak et al.^29^ proposed a strut-and-tie model (STM) for predicting the shear strength of squat shear walls subjected to seismic loading. Their model considered the combined action of struts and ties, enabling a more accurate representation of the internal force transfer mechanisms. Compared to traditional methods such as ACI 318-19,^30^ the proposed model demonstrated enhanced predictive accuracy, particularly in walls with complex reinforcement layouts. Their research underscores the significance of web reinforcement and the appropriate design of boundary elements to ensure an improved shear performance and reduced crack propagation.

Building upon these foundational studies, the present research investigates hybrid reinforcement configurations combining steel and FRP bars in squat shear walls under blast loading conditions. The integration of FRP and steel reinforcements leverages the tensile strength, stiffness, and corrosion resistance of FRPs while preserving the ductility and energy absorption characteristics of the steel. This hybrid approach aims to optimize shear capacity, enhance energy dissipation, and control crack development under extreme loads, offering a novel perspective on blast-resistant RC wall design. By bridging the knowledge gap between seismic and blast performance, this study provides a comprehensive evaluation of hybrid-reinforced shear walls, contributing to the advancement of blast-resistant infrastructure design in civil engineering practice.

In order to address these challenges, researchers have increasingly focused on advanced composite materials such as fiber-reinforced polymers (FRPs), which exhibit superior mechanical properties, including high tensile strength, corrosion resistance, and favorable strength-to-weight ratios.^8^ Among the commonly used FRPs, glass fiber-reinforced polymer (GFRP), carbon fiber-reinforced polymer (CFRP), and basalt fiber-reinforced polymer (BFRP) have demonstrated significant potential in structural applications. GFRP is valued for its cost-effectiveness and corrosion resistance, while CFRP offers exceptional tensile strength and stiffness, making it ideal for high-performance applications.^14^ BFRP, derived from natural basalt rock, provides an environmentally sustainable alternative with comparable strength and durability.^25^

Despite considerable progress in using individual FRP types, limited studies have explored their combined use with conventional steel reinforcements in hybrid configurations, particularly in blast-resistant applications. The integration of FRPs and steel reinforcements in a single structural system offers the potential to combine the best properties of both materials—leveraging the tensile capacity and corrosion resistance of FRPs while maintaining the ductility and energy absorption characteristics of steel.^1^ Additionally, while many studies focus on seismic performance, the dynamic behavior of hybrid-reinforced RC shear walls under blast-induced loads remains underexplored, highlighting a critical research gap.^24^

This study aims to bridge this gap by investigating the blast resistance of RC shear walls reinforced with various hybrid configurations of steel and FRP bars. Unlike previous research limited to single-reinforcement systems, this work evaluates multiple reinforcement patterns to determine optimal configurations that maximize the blast resistance, energy absorption, and structural resilience. A validated finite element model (FEM) was employed, ensuring accurate numerical simulations based on experimental benchmarks from relevant literature.^31^ The study also provides practical design recommendations for engineers and researchers seeking to improve the blast resistance of RC shear walls, contributing to a safer, more resilient infrastructure.

## Numerical Analysis

This section outlines the numerical analysis conducted to evaluate the blast resistance of reinforced concrete (RC) shear walls reinforced with different fiber-reinforced polymer (FRP) and steel configurations. The analysis involved the validation of the finite element model (FEM) against experimental data and numerical simulations using advanced computational techniques.

### Numerical Model Validation

The validation of the finite element model (FEM) is a crucial step in ensuring an accurate simulation of structural behavior under extreme dynamic loading conditions. In this study, the experimental research conducted by Li et al.^26^ was used as the primary reference for validating the FEM due to its comprehensive analysis of reinforced concrete slabs subjected to contact explosions. Their experiments provided detailed measurements of key structural damage metrics, including spalling diameters, crater formation, and deformation patterns, offering a reliable benchmark for numerical verification.

**Figure 1:**
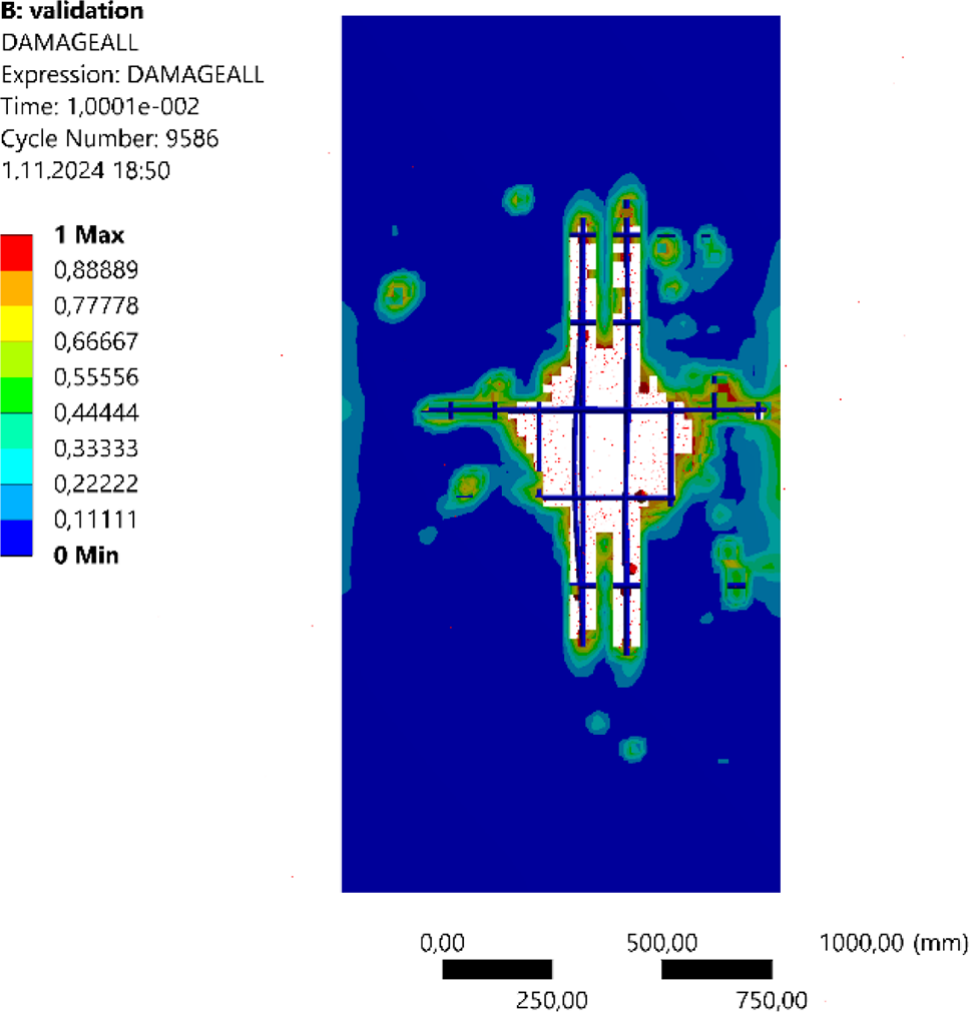
Validation model.

To ensure the accuracy and reliability of the finite element analysis (FEA) model developed for this study, a numerical validation was conducted based on established experimental and numerical data from previous research on RC slabs under explosive loads. The study by Zhao et al.,^32^ which investigates the damage characteristics of RC slabs subjected to air and underwater contact explosions, served as another reference for the validation process. This research employed three advanced numerical methods, coupled Eulerian–Lagrangian (CEL), smoothed particle hydrodynamics (SPH), and a hybrid FEM-SPH approach, to simulate blast-induced damage in RC slabs. Among these methods, the FEM-SPH hybrid approach was validated against experimental results, demonstrating effective predictions of crater formation, spalling, and overall failure modes, making it highly suitable for simulating localized dynamic responses in RC structures subjected to blast loads. The validation model is illustrated in Figure 1, providing a visual representation of the setup used to align with the parameters and conditions established. The analyses in this study were performed using ANSYS software, a robust finite element analysis tool widely used for simulating complex structural behaviors under dynamic and static loading conditions.^33^

A comprehensive mesh independence study was conducted using various mesh densities (10, 15, 20, 25, and 30 mm) to determine the optimal mesh size that balances computational efficiency and simulation accuracy. Based on the analysis, it was determined that results converged at a mesh size of 20 mm, which was subsequently used for all numerical simulations, as it provided an optimal balance between computational efficiency and accuracy. Critical regions, such as areas with high stress concentrations and sharp geometric features, were refined to a higher resolution. Furthermore, the explosive region was discretized with a finer mesh size of 5 mm to ensure an accurate representation of the dynamic behavior during the initial stages of the explosion. The study also emphasized that reducing the mesh size further would yield diminishing returns in accuracy while significantly increasing the computational costs.

Concrete was modeled with temperature- and strain-rate-dependent mechanical properties to reflect its behavior under high temperatures and dynamic loading conditions. The reinforcement steel was modeled using an elastic-plastic approach, incorporating dynamic increase factors (DIF) to account for strain-rate effects. The explosive was simulated using the smoothed particle hydrodynamics (SPH) method to accurately capture the interaction between the blast wave and the structure.

Boundary conditions were defined by fully fixing the base of the models while restricting lateral and top displacements. The applied loads were simulated as time-dependent pressure distributions to replicate real-world conditions accurately. These modeling approaches and refinements have enhanced the reliability of the analyses, with the numerical results showing excellent agreement with the experimental data.

**Figure 2:**
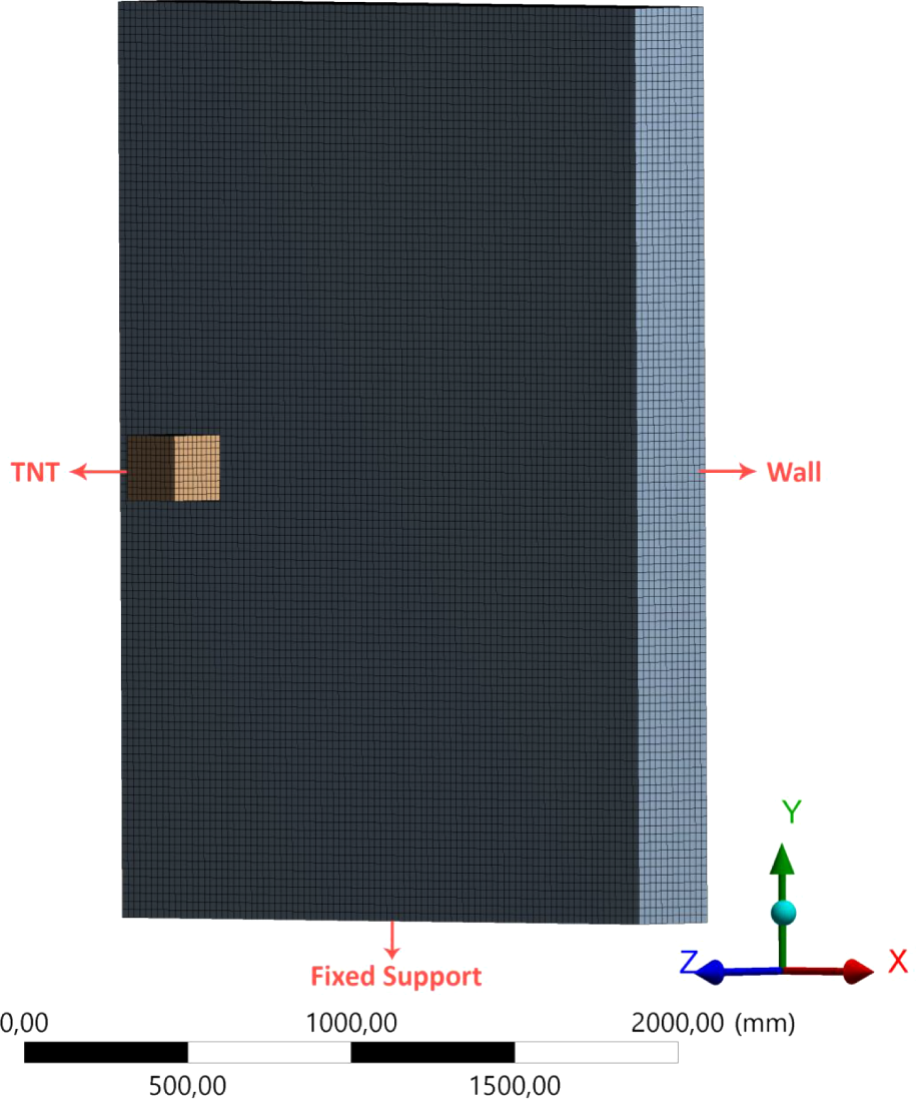
Mesh view of the analysis model for the shear wall.

A mesh size of 20 mm was adopted for the concrete and rebars after several mesh configurations. This mesh configuration, along with the FEM-SPH framework, enabled a detailed representation of blast impact and subsequent material deformations and provided a suitable compromise, ensuring high-resolution damage prediction while maintaining manageable computational demands. This configuration also ensured that the results were both accurate and computationally feasible, supporting reliable simulation outcomes for damage assessment and performance evaluation of the structural components under dynamic loads. The mesh view of the analysis model is provided in Figure 2.

The numerical model parameters were also verified by comparing the numerical results to experimental findings from previous studies. The FEM validation process involved replicating essential experimental conditions, such as material properties, boundary conditions, and blast load characteristics, following the methodologies described by Li et al.^26^ and Zhao et al.^32^ Critical damage indicators, including crater dimensions, spalling patterns, tensile cracking, and concrete crushing, were directly compared with the numerical and experimental results. The close agreement between these results confirmed the accuracy, stability, and predictive capability of the FEM model used in these publications.

The numerical techniques implemented in this study were cross-validated with findings from advanced FEM simulations of concrete members under extreme loads conducted in previous publications. This dual-validation approach, combining experimental benchmarking and numerical comparison, ensured that the selected parameters produced accurate simulations, including realistic damage propagation, crack development, and failure patterns under explosive loading conditions.

By establishing this validation benchmark, the present study ensures that the developed model accurately represents the complex behaviors of reinforced concrete under high-strain, high-energy impact conditions. This foundation allows for a robust investigation into the blast resistance of hybrid FRP-reinforced concrete structures, providing a reliable framework for exploring performance under extreme loading.

### Numerical Model Simulation

Following the validation of the numerical model, detailed analyses were conducted on a reinforced concrete shear wall with dimensions of 2100 mm in height, 2800 mm in width, and 300 mm in thickness. The numerical model employed the concrete damage plasticity (CDP) model to represent concrete’s nonlinear behavior under compressive and tensile stresses. Steel reinforcements were modeled by using elastoplastic material definitions with strain-hardening properties. FEM simulations were further enhanced by employing the smoothed-particle hydrodynamics (SPH) technique to simulate blast wave propagation and structural interactions.

The model was designed to examine the performance of various reinforcement configurations under blast loading with systematic variations in reinforcement type. Specifically, ten different configurations, combining longitudinal and transverse reinforcements made of traditional steel (rebar), basalt fiber reinforced polymer (BFRP), carbon fiber reinforced polymer (CFRP), and glass fiber reinforced polymer (GFRP), were analyzed to assess blast resistance and energy dissipation.

The FEM simulations were performed using ANSYS software, applying a mesh size of 20 mm to balance the computational efficiency and simulation accuracy. Boundary conditions included fixed constraints at the base of the wall, allowing free expansion of other faces. The blast load was modeled as a time-dependent pressure wave using established empirical equations. Material properties were defined according to experimental data and the relevant literature. The compressive strength of concrete was set at 35 MPa, while the yield strength of the steel reinforcements was 500 MPa. Mechanical properties for CFRP, GFRP, and BFRP were assigned based on values reported in the literature, ensuring an accurate representation of the reinforcement materials during blast loading simulations. Simulation results revealed detailed structural responses, including displacement, energy absorption, and surface damage patterns. CFRP-reinforced walls exhibited superior blast resistance due to CFRP’s high tensile strength and stiffness. BFRP configurations demonstrated improved energy dissipation, although with greater deformation, while GFRP provided a balance between flexibility and structural stability. These findings highlight the potential of hybrid reinforcement systems for enhancing blast resistance in RC shear walls, offering valuable design insights for future structural engineering applications.

**Figure 3:**
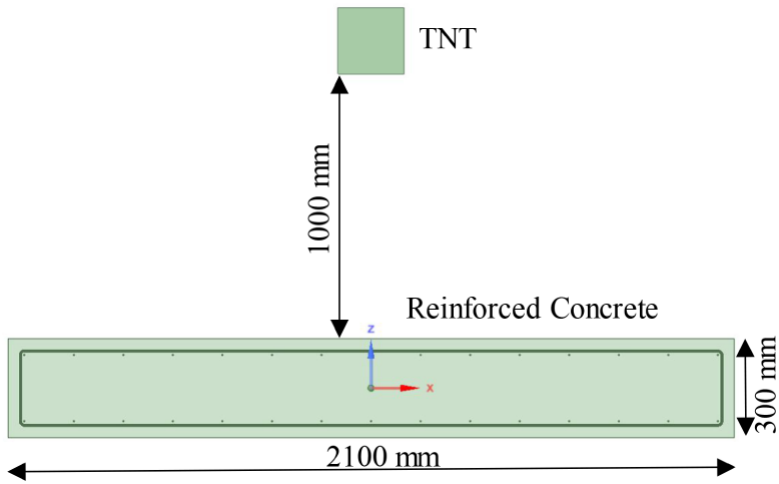
Distance of explosive from shear wall.

**Figure 4:**
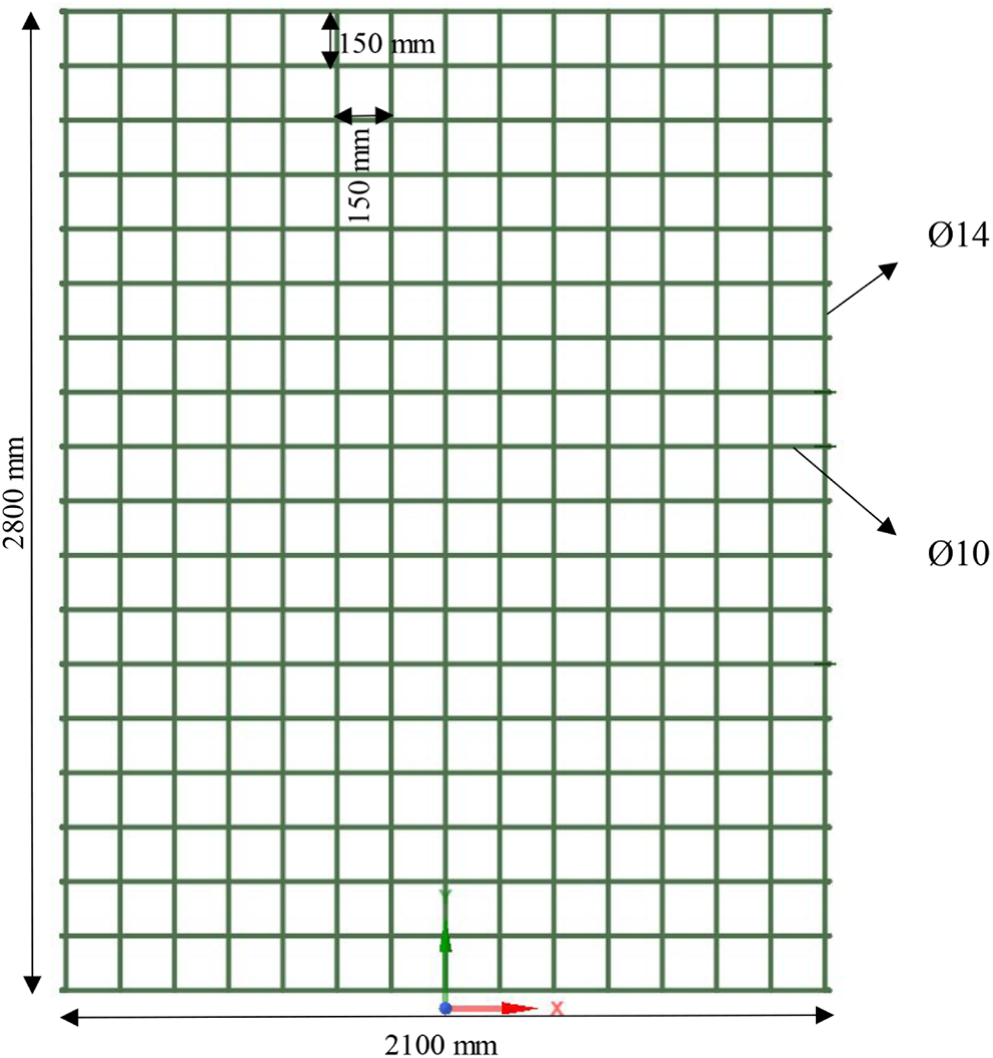
Reinforcement details of the shear wall configurations.

The distance between the explosive and shear wall is shown in Figure 3, while the reinforcement details are provided in Figure 4. Explosives were placed at the center of the shear wall to achieve a central contact explosion in the test setup. Relevant studies indicate that the damage index of the wall decreases as the explosive is moved gradually from the center toward the edges. Therefore, only the midspan contact explosion, which causes maximum damage to the wall, is considered in this study. The explosives were stacked using standard TNT explosive blocks. Each standard TNT block, measuring 100 mm in length, 50 mm in width, and 25 mm in height, has a mass of 200 g and a density of 1.6 g/cm^3^. Based on the previous studies, the material properties assigned to the RC shear wall model used in the analysis are provided in Table 1.

**Table 1:** Structural Material Properties

Type	Isomorphic model	ρ (kg/m^3^)	*E* (GPa)	ν
Concrete	Plastic damage modeling	2200	36.3	0.20
Steel	Elasticity	7850	200.0	0.30
CFRP	Elasticity	1600	140.0	0.27
GFRP	Elasticity	1700	40.2	0.25
BFRP	Elasticity	2050	61.0	0.30

The longitudinal reinforcements were modeled with a diameter of 14 mm, and the transverse reinforcements had a diameter of 10 mm. Both types of reinforcement were spaced at 150 mm intervals to ensure a consistent load distribution and structural integrity. The reinforcement configurations applied in the model are as follows:

Configuration 1: All reinforcements (both longitudinal and transverse) are traditional steel (rebar).

Configuration 2: Longitudinal reinforcements are BFRP, while transverse reinforcements are traditional steel.

Configuration 3: Transverse reinforcements are BFRP, while longitudinal reinforcements are traditional steel.

Configuration 4: Both longitudinal and transverse reinforcements are entirely BFRP.

Configuration 5: Longitudinal reinforcements are CFRP, while transverse reinforcements are traditional steel.

Configuration 6: Transverse reinforcements are CFRP, while longitudinal reinforcements are traditional steel.

Configuration 7: Both longitudinal and transverse reinforcements are entirely CFRP.

Configuration 8: Longitudinal reinforcements are GFRP, while transverse reinforcements are traditional steel.

Configuration 9: Transverse reinforcements are GFRP, while longitudinal reinforcements are traditional steel.

Configuration 10: Both longitudinal and transverse reinforcements are entirely GFRP.

These specific configurations were chosen to evaluate the individual and combined effects of different FRP materials on the structural performance, focusing on blast resistance, energy absorption, and deformation characteristics. The model drawings for each reinforcement configuration are provided in Figure 2, visually detailing the arrangement and layout of reinforcements within the RC shear wall for reference and clarity. You can use the naming convention (e.g., Configurations 1, 2, etc.) to label the analysis images accordingly.

### Numerical Model Outcomes

Energy absorption, transmission, and dissipation in RC shear walls subjected to blast loading are evaluated using advanced finite element modeling integrated with the smoothed particle hydrodynamics method. This approach enables accurate tracking of energy flow throughout the structure by monitoring key performance indicators, such as internal energy accumulation, wave propagation, and material failure mechanisms.

Energy absorption is measured by calculating the total internal energy stored within the RC wall during blast-induced deformations. This energy reflects the strain energy generated through elastic and plastic deformations, as the structure resists blast loads. The FEM model records this energy as it accumulates in the concrete matrix and reinforcement bars, particularly during the critical phases of tensile cracking and compressive yielding.

Energy transmission is assessed through wave propagation analysis, where blast-induced shock waves travel through the structure’s cross-section. The transmission capacity depends on the mechanical properties of materials, such as the modulus of elasticity and tensile strength of steel and FRP reinforcements. The numerical simulations track how energy propagates from the point of blast impact through the wall, focusing on stress wave reflections and their interactions with boundaries and reinforcements.

Energy dissipation is determined by evaluating the energy lost due to inelastic behaviors such as plastic deformation, crack propagation, and spallation of concrete. FEM-SPH coupling facilitates accurate modeling of these dissipative mechanisms, allowing large deformations and localized failures to be captured without numerical instability. The dissipation process includes damage from tensile cracking in the concrete, yielding of steel and FRP reinforcements, and fracture-induced energy loss, contributing to the overall energy absorption and structural resistance.

## Results and Discussion

### Displacement Analysis Integration

**Figure 5:**
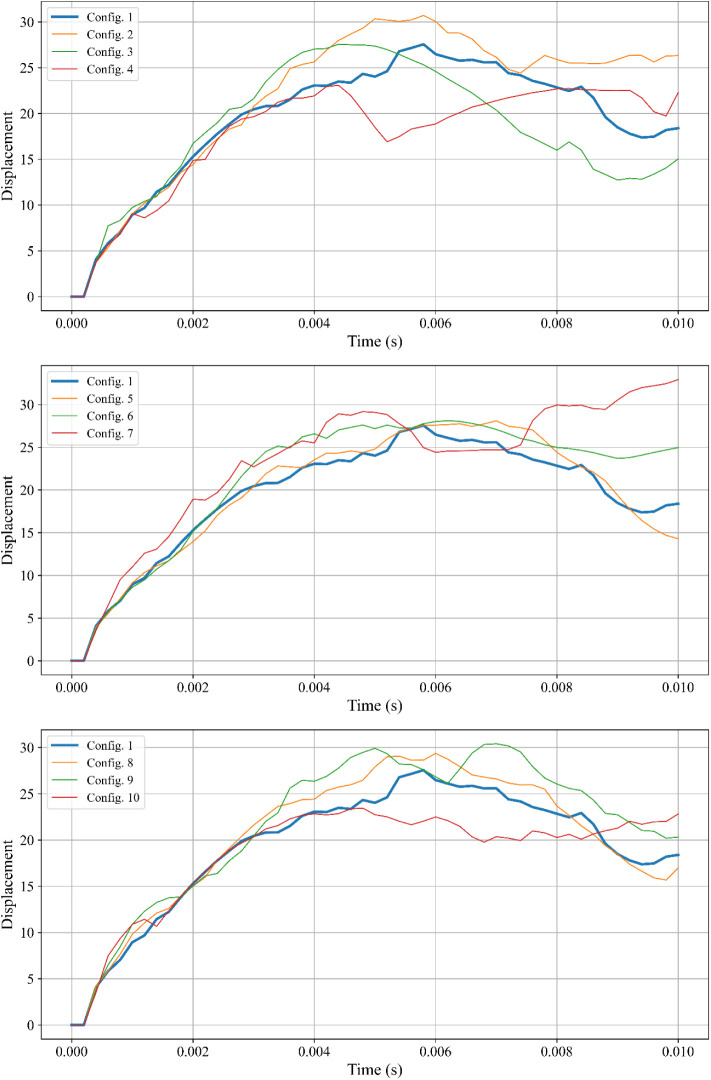
Displacement-time graph for all configurations under blast loading: Configurations 1–4 (steel and BFRP); Configurations 5–7 (CFRP); Configurations 8–10 (GFRP).

The displacement-time graphs provided for different reinforcement configurations (Configurations 1–10) are shown in Figure 5, offering a detailed understanding of the RC shear wall’s deformation behavior under blast loading. Displacement is a critical parameter that reflects the structural response, energy dissipation, and flexibility of the wall.

The numerical simulations conducted in this study demonstrated that CFRP-reinforced shear walls exhibited the least displacement under blast loading due to the material’s high tensile strength and stiffness. This result aligns closely with the findings of Zhao et al.,^32^ who observed reduced deformation in RC slabs reinforced with high-strength composite materials under dynamic blast conditions. Additionally, GFRP and BFRP-reinforced configurations in the present study displayed larger displacements, consistent with Hosseini et al.,^19^ who reported that GFRP reinforcements improved energy absorption while compromising structural stiffness due to their lower elastic modulus.

#### Configurations 1–4 (Steel and BFRP Reinforcements)

Configuration 1, utilizing traditional steel (rebar) as both longitudinal and transverse reinforcement, displays moderate displacement throughout the simulation. The stiffness of steel ensures a balanced response with controlled deformation and adequate energy absorption.

Configurations 2 and 3, which incorporate BFRP partially (longitudinal or transverse), show increased displacement compared to Configuration 1. Specifically, Configuration 2 (BFRP in longitudinal reinforcements) exhibits higher peak displacement due to BFRP’s lower stiffness compared to steel, resulting in greater flexibility but reduced resistance to deformation. Similarly, Configuration 3 (BFRP in transverse reinforcements) follows a similar trend, but its peak displacement is slightly lower than Configuration 2 due to steel’s contribution in the longitudinal direction.

Configuration 4, where both longitudinal and transverse reinforcements are BFRP, shows the highest displacement among these configurations. The complete substitution of steel with BFRP increases flexibility significantly but at the cost of reduced stiffness, causing larger deformations during the blast impact.

#### Configurations 5–7 (CFRP Reinforcements)

CFRP-based configurations exhibit superior performance due to CFRP’s high tensile strength and stiffness. Configurations 5 (CFRP in longitudinal reinforcements) and 6 (CFRP in transverse reinforcements) demonstrate moderate displacement, lower than Configurations 2–4, indicating better blast resistance. Configuration 7, which uses CFRP for both longitudinal and transverse reinforcements, shows the least displacement overall. This configuration highlights CFRP’s ability to resist blast-induced forces effectively while maintaining the structural integrity of the wall. The minimal displacement observed in Configuration 7 demonstrates that CFRP is an ideal material for applications requiring high resistance to dynamic loads.

#### Configurations 8–10 (GFRP Reinforcements)

GFRP configurations show intermediate behavior between BFRP and CFRP. Configurations 8 (GFRP in longitudinal reinforcements) and 9 (GFRP in transverse reinforcements) display displacement trends similar to Configuration 1, with slightly higher peak values, indicating a balance between stiffness and flexibility. Configuration 10, with full GFRP reinforcement, exhibits higher displacement levels, similar to those of Configuration 4 (full BFRP). This suggests that while GFRP offers better stiffness than BFRP, it still results in greater deformation compared to steel or CFRP.

### Surface Damage Assessment

**Figure 6:**
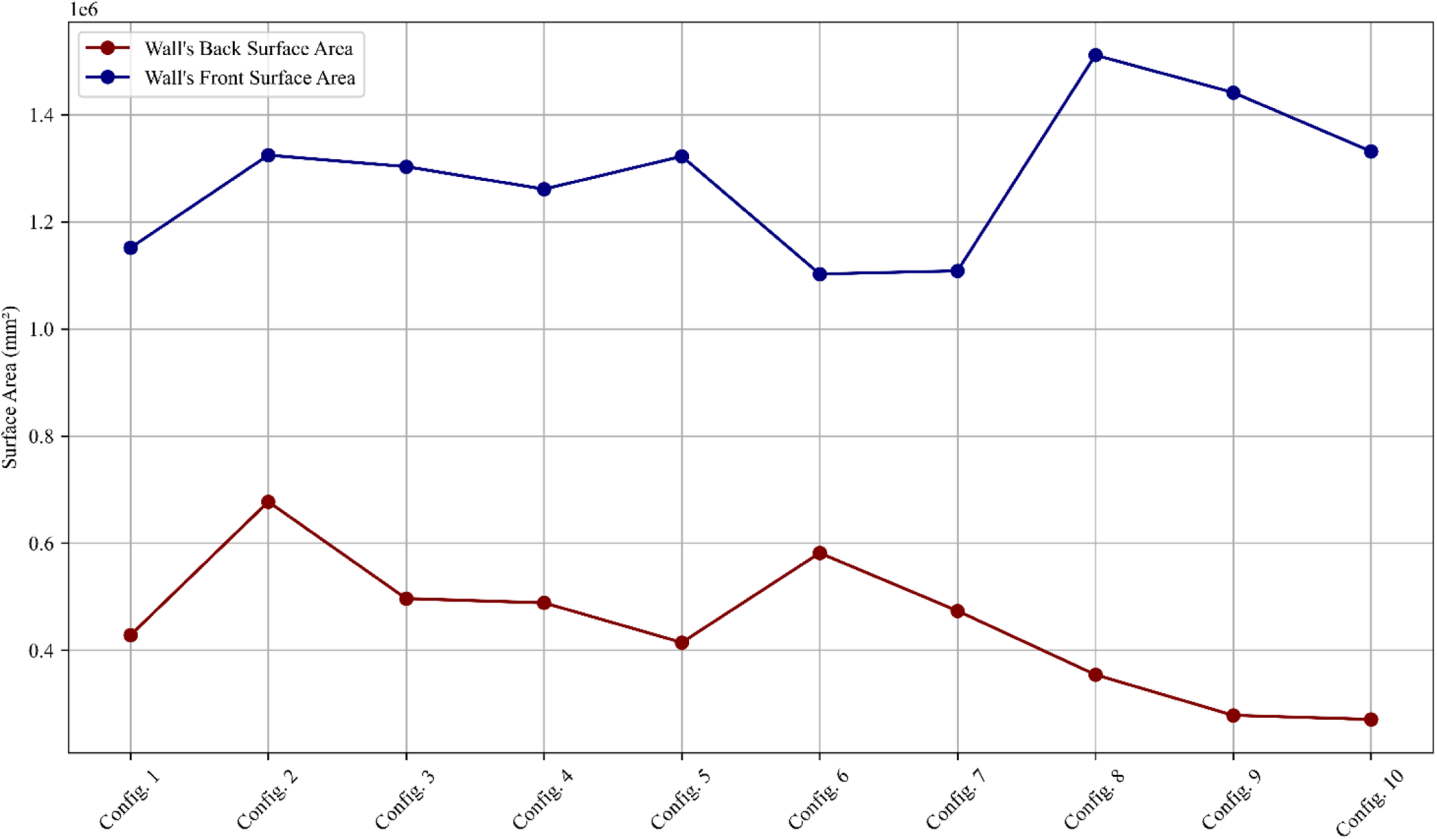
Surface area damage (front and back) for all configurations under blast loading.

**Figure 7:**
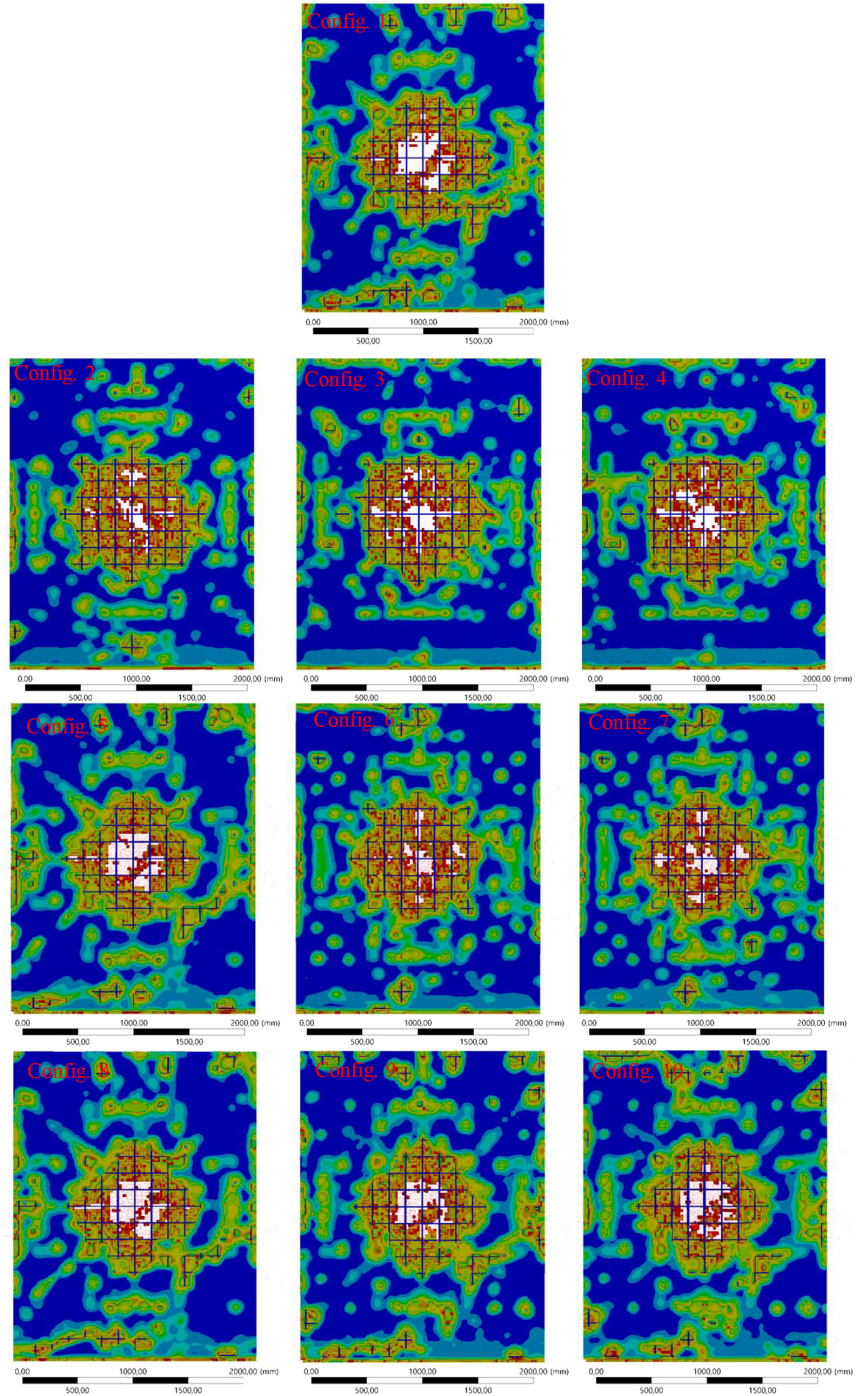
Front surface damage visualization for all configurations under blast loading.

**Figure 8:**
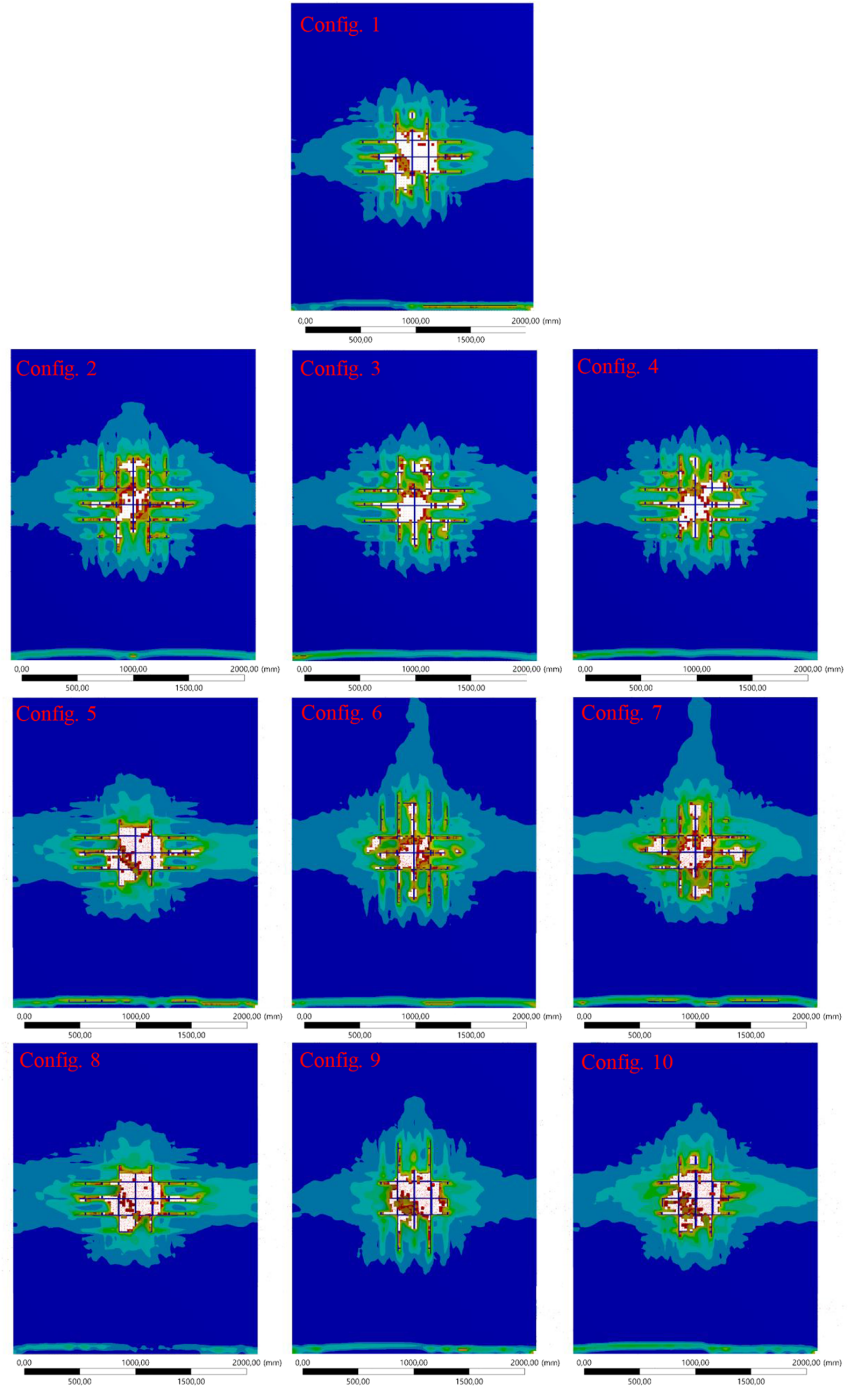
Back surface damage visualization for all configurations under blast loading.

The surface area damage graph, shown in Figure 6, evaluates the extent of blast-induced damage on the front (blast-facing) and back (opposite) surfaces of the wall for all configurations. Surface damage is a critical indicator of the wall’s ability to absorb and distribute blast energy while maintaining structural integrity. Detailed damage visualizations of the front and back surfaces of the RC shear wall for all configurations are presented in Figures 7 and 8, respectively.

The simulated results showed that walls fully reinforced with CFRP experienced significantly reduced surface damage, including minimal cracking and spalling, compared to configurations using BFRP and GFRP. This is supported by Pavlović and Radović,^31^ who found that CFRP-reinforced concrete demonstrated superior impact resistance due to its high tensile strength and effective crack-bridging capabilities. Similarly, Tanapornraweekit and Chatupote^34^ observed that CFRP reinforcement substantially reduced blast-induced surface damage by limiting tensile failure and preventing large-scale material loss, findings that align with the current study’s numerical outcomes.

#### Front Surface Area (Figure 7)

The front surface experiences direct exposure to the blast, resulting in significant damage. Configuration 1 (pure rebar) shows moderate damage, reflecting the balanced energy dissipation and resistance provided by traditional steel.

Configurations 5–7 incorporating CFRP display the smallest affected areas on the front surface. Configuration 7, in particular, demonstrates the least damage due to CFRP’s high strength and stiffness, which effectively disperses the blast energy and prevents localized deformation.

In contrast, configurations with BFRP (Configurations 2–4) and GFRP (Configurations 8–10) show larger affected areas, particularly Configurations 4 and 10, where full substitution of steel results in lower resistance to concentrated blast forces. These configurations highlight the trade-off between flexibility and surface damage when these materials.

#### Back Surface Area (Figure 8)

Damage to the back surface indicates the extent of the energy transmission through the wall. Configuration 1 shows moderate back surface damage, consistent with its performance on the front surface. CFRP-based configurations (Configurations 5–7) exhibit the least back surface damage, particularly Configuration 7, which effectively limits the energy transfer due to CFRP’s superior material properties.

BFRP and GFRP configurations (Configurations 2–4 and 8–10) show greater back surface damage, with Configurations 4 and 10 experiencing the most significant damage. This is attributed to their higher flexibility, which allows for greater energy transmission through the wall, resulting in an amplified deformation at the back.

### Energy Absorption and Dissipation Metrics

The numerical simulations indicated that hybrid reinforcement configurations achieved superior energy absorption and dissipation due to the combination of materials with complementary mechanical properties. He et al.^23^ highlighted similar results, showing that hybrid FRP systems maximized energy dissipation by balancing stiffness and flexibility. In particular, fully CFRP-reinforced walls in the present study displayed high energy absorption capacity, consistent with Hosseini et al.,^35^ who demonstrated that CFRP effectively resists dynamic loads by maintaining structural stability while dissipating impact energy through controlled material deformation.

### Model Validation Comparisons

The validation of the FEM developed in this study closely matched the experimental damage profiles reported by Zhao et al.,^32^ including spalling, cracking, and crater formation observed under contact explosion scenarios. The FEM-SPH coupling technique employed in this research successfully captured these damage characteristics, reinforcing the validity of the model for simulating complex blast-structure interactions. The high correlation between numerical and experimental results highlights the model’s applicability for evaluating various reinforcement configurations, supporting its use in future performance assessments of RC shear walls.

## Conclusions

This study provides a comprehensive analysis of the blast resistance of reinforced concrete shear walls with various reinforcement configurations, focusing on the effects of hybrid materials, such as BFRP, CFRP, and GFRP, alongside traditional steel (rebar). The findings demonstrate the trade-offs between stiffness, energy dissipation, and surface damage across configurations and highlight the potential of advanced fiber-reinforced polymer (FRP) reinforcements in blast-resistant structural applications. Key conclusions drawn from this work are outlined below:

### Effectiveness of CFRP as a Reinforcement Material

Configurations with CFRP, especially Configuration 7 (full CFRP reinforcement), consistently exhibited superior performance in terms of minimizing displacement and surface damage. The high tensile strength and stiffness of CFRP enable it to effectively resist blast-induced forces, reducing deformation and preventing significant damage to both the front and back surfaces of the shear wall. CFRP configurations showed the least energy transmission through the wall, as evidenced by the minimal back surface damage. This highlights CFRP’s potential for applications requiring high structural integrity under extreme dynamic loading conditions.

### Performance of BFRP in Blast-Resistant Applications

Configurations utilizing BFRP (Configurations 2–4) demonstrated higher energy absorption capabilities due to the material’s flexibility. However, this flexibility led to increased displacement and more significant surface damage, particularly when BFRP completely replaced traditional steel (Configuration 4). The trade-off between energy dissipation and stiffness makes BFRP suitable for applications where flexibility and corrosion resistance are more critical than minimizing structural deformation, such as in marine or coastal environments.

### Intermediate Behavior of GFRP

GFRP configurations (Configurations 8–10) displayed performance characteristics between BFRP and CFRP. While Configuration 10 (full GFRP reinforcement) resulted in higher displacement and surface damage than CFRP, it performed better than BFRP, making it a viable option for cost-effective reinforcement in moderately demanding applications. Partial replacement of steel with GFRP (Configurations 8 and 9) provided a balance between stiffness and energy dissipation, offering an economical solution for scenarios where moderate blast resistance is sufficient.

### Role of Hybrid Configurations

Hybrid configurations, where steel was partially replaced by FRP materials (e.g., Configurations 2, 3, 8, and 9), demonstrated a balanced performance by combining the stiffness of steel with the flexibility of FRP. These configurations reduced surface damage and displacement to acceptable levels while maintaining cost efficiency. Hybrid reinforcements are recommended for situations requiring an optimized balance between performance and material cost such as retrofitting existing structures or constructing secondary blast-resistant components.

### Impact of Reinforcement Material on Displacement

The displacement analysis revealed that steel-based configurations (Configuration 1) provided moderate deformation control, while CFRP significantly reduced the displacement. In contrast, BFRP and GFRP configurations resulted in higher deformation due to their lower stiffness. The ability of CFRP to restrict displacement positions it as the most suitable material for high-impact scenarios, whereas BFRP and GFRP are more appropriate for cases prioritizing energy absorption.

### Surface Damage Considerations

The surface damage analysis showed that CFRP configurations (Configurations 5–7) minimized the affected areas on both the front and back surfaces of the wall, demonstrating their effectiveness in reducing localized energy transmission. Conversely, BFRP and GFRP configurations, particularly when used exclusively (Configurations 4 and 10), exhibited greater damage areas, indicating the need for improved design strategies when these materials are used.

### Practical Implications

In terms of the design strategies, considering high-risk structures, such as military installations or critical infrastructure, full CFRP reinforcement (Configuration 7) should be prioritized due to its exceptional blast resistance and structural stability.

### Limitations and Future Research

Material limitations: while CFRP demonstrated superior performance, its higher cost and potential brittleness under certain conditions require further investigation. Similarly, the flexibility of BFRP and GFRP may limit their applications in scenarios demanding high stiffness.

Numerical model enhancements: future studies should incorporate more complex loading scenarios, such as multidirectional blasts, to simulate real-world conditions more accurately. Long-term durability: research should also examine the long-term durability of FRP-reinforced structures under combined environmental and dynamic loading conditions, including temperature and humidity effects.

Experimental validation: while this study relies on numerical simulations, experimental validation of the findings would provide further confidence in the applicability of these materials in practical scenarios. CFRP (Configuration 7): best suited for critical infrastructure requiring high blast resistance. Hybrid configurations: optimal for balancing cost and performance, particularly Configurations 2 and 8. BFRP and GFRP: suitable for environments prioritizing energy dissipation and corrosion resistance, with Configurations 4 and 10 requiring additional design considerations for reducing deformation and surface damage.
